# Oral and Dental Sequelae After Oncological Treatment in Children: A Systematic Review

**DOI:** 10.3390/jcm14155479

**Published:** 2025-08-04

**Authors:** Lidia Torrecillas-Quiles, Inmaculada Gómez-Ríos, Irene Jiménez-García, Ildefonso Serrano-Belmonte, Antonio José Ortiz-Ruiz, Clara Serna-Muñoz

**Affiliations:** 1 Department of Integrated Pediatric Dentistry, Faculty of Medicine, University of Murcia, 30100 Murcia, Spain; lidia.torrecillasq@um.es (L.T.-Q.); ajortiz@um.es (A.J.O.-R.); clara.serna@um.es (C.S.-M.); 2 Sección de Oncohematología Pediátrica, Hospital Clínico Universitario Virgen de la Arrixaca, 30120 Murcia, Spain; irene.jimenez@carm.es; 3 Department of Integrated Prosthodontics, Faculty of Medicine, University of Murcia, 30100 Murcia, Spain; iserrano@um.es; 4 Biomedical Research Institute of Murcia Pascual Parilla-IMIB, 30120 Murcia, Spain

**Keywords:** childhood cancer, oncological treatment, dental sequelae, systematic review

## Abstract

**Background**: Childhood cancer is considered one of the main causes of mortality and morbidity worldwide. There is strong evidence of the oral toxic effects of oncologic treatments, but their incidence is difficult to determine. The novel therapeutic strategies in Pediatric Oncology have led to increased survival in this population, resulting in an increased incidence of long-term effects, which diminish the patient’s quality of life. **Methods**: The search for articles started on 5 November 2024 and ended on 5 December 2024. Following the PRISMA Statement, a total of 1266 articles were obtained, from which 13 were selected for review. All articles were considered to be of high quality. The antineoplastic treatments used in them were chemotherapy, radiotherapy, surgery and immune therapy. **Results**: Most articles were cohorts and case controls. Only one case report was obtained. The results revealed that the most prevalent sequelae in the pediatric population after antineoplastic treatment were enamel alterations, microdontia, dental caries, periodontal disease, gingivitis, hyposalivation, alteration of the oral microbiome, alteration of mandibular bone density and malocclusion. The lesions are different depending on the therapy used. **Conclusions**: Oncologic treatments in children with cancer cause multiple oral sequelae such as microdontia, dental caries, enamel alterations, salivary gland alterations, mucositis and root resorption. It cannot be concluded which therapy has the most detrimental effect as each has a different mechanism of action in the oral cavity.

## 1. Introduction

At present, childhood cancer has become one of the main causes of mortality worldwide. Each year, a total of 29,000 children are diagnosed with cancer, according to values provided by the World Health Organization (WHO), and there is no relationship between sex and age [1].

Although childhood cancer continues to be the leading cause of mortality in children in our environment, advances in treatments have managed to increase overall survival up to 80% at 5 years in Spain [2]. Treatments of pediatric cancers present unique challenges compared to adult cancers due to the harsh side effects of such treatments, including chemo, radio and immunotherapy, as well as hematopoietic stem cell transplantation [3]. It is also known that the use of these therapies causes long-term side effects, including oral sequelae. The mechanisms that cause these sequelae are not clearly understood. But it is known that they can affect the process of odontogenesis, so any change caused by antineoplastic treatment during that period (from 6 months of age to 12 years) can affect dental development [4,5,6].

Several risk factors are attributed to the occurrence of oral sequelae, including patient age, type of cancer, dose and intensity of treatment [1]. The age of the patient is one of the most relevant factors, since the younger the age of the patient, the greater tends to be the severity of its sequelae due to the continuous growth development of the individual [1,4,7,8].

The oral sequelae can be grouped into three groups according to the tissue affected: alterations affecting the dental structure, soft tissue involvement and bone defects.

Among the dental alterations that most affect child patients are dental caries, enamel defects, microdontia, root abnormalities and alterations in the dental maturation process. Of these alterations, the most frequent are microdontia and dental caries. Microdontia is a consequence of the alterations that occur in the course of odontogenesis due to antineoplastic therapy and specifically affects the upper first premolars, upper second molars and lower second molars [7]. Dental caries is mainly related to enamel demineralization and decreased salivary pH during antineoplastic therapy and affects teeth that are erupted at that time [9]. Alteration of the salivary glands produces also a decrease in salivary flow and xerostomia, which favors the appearance of dental caries. However, this salivary function can be recovered 12 to 18 months after the cessation of treatment, depending on the dose of treatment and the area treated [10].

Regarding soft tissue involvement, oral mucositis and salivary gland alteration, among others, are relevant. Oral mucositis is one of the most relevant clinical manifestations in patients undergoing chemotherapy and head and neck radiotherapy [10,11].

Osteonecrosis is the most important alteration that affects bone tissue. This condition is relevant in patients with acute lymphoblastic leukemia, and its incidence has increased in recent decades by up to 72%. Therefore, prevention is very important in these patients to avoid risk and long-term complications such as reversion [12].

By virtue of the above, it has been demonstrated that oncological treatment can generate oral sequelae that can affect the health and well-being of child patients. Therefore, preventive oral health and early intervention are essential to stabilize the risk of oral lesions and their severity [13,14].

Following the PICO question, the objective of this systematic review was formulated:

In patients who had childhood cancer (0–14 years) and received oncological treatment (chemotherapy, radiotherapy, immunotherapy and surgery), how do these therapies affect the appearance of oral and dental sequelae, compared to healthy children without a history of cancer?

- P (patient, problem, population): patients who had cancer during their childhood from 0 to 14 years of age and have received treatment.
- I (intervention): use of different therapies for the treatment of childhood cancer (chemotherapy, radiotherapy, immunotherapy).
- C (comparison, control): childhood cancer-free patients aged 0–14 years.
- O (outcome): oral and dental sequelae after treatment.

## 2. Methods

This review was registered in PROSPERO (CRD420251023914), and the search was performed following the PRISMA (Preferred Reporting Items for Systematic Review and Meta-analysis) guidelines [15] in the primary databases MEDLINE (Pubmed), Scopus, Web of Science and Liliacs. The review was initiated on 5 November 2024 and completed on 5 December 2024.

The following search strategy was used: (((“Childhood cancer” OR “Neoplasm”) AND (((“Pediatric Dentistry” OR “Dental Care” OR “Tooth” OR “Dental Enamel” OR “Dental Caries” OR “Mucositis” OR “Osteonecrosis” OR “gingivitis”)))) AND ((“Treatment” OR “Drug Therapy” OR “Radiotherapy” OR “Surgery” OR “immunotherapy”)))) AND children NOT (Adult) “. Only articles published between 2014 and 2024 were selected. Randomized controlled trials (RCTs), case control studies, retrospective observational studies, cross-sectional studies, case report articles, case series articles, and in vivo and in situ human clinical trials that meet the PICO criteria for outcome measures were included.

The references obtained were directed to the ZOTERO citation manager, where duplicates were eliminated. After their elimination, a screening was performed, with the selection of articles by title and after the abstracts. Pilot studies, systematic reviews, letters to the editor, book chapters, animal studies, in vitro studies and opinion articles were excluded.

Those articles that met the inclusion criteria were analyzed for admission and synthesis through full-text screening. Data extraction was performed by two authors independently (L.T. and C.S.). If there was any disagreement between the two authors, a third party (A.J.O.) was consulted.

The data sought related to the possible side effects of cancer treatment in children: alterations in shape, root, salivary flow, caries, mucositis and orthodontics alteration.

The quality assessment of the articles was carried out using different scales according to the type of article. The Newcastle-Ottawa scale (NOS) was used for cohort, cross-sectional and case control articles. This scale contains 8 items and rates quality from 0 to 9 stars. High-quality articles are those with a value equal to or higher than 8. In this case, it only gave a maximum value of 8 stars because in the “Comparability” section, we only assigned one of the questions. Therefore, high-quality articles are considered to be those with a score of 7 or more stars. The CARE GUIDE (CAse REport) was used for the analysis of a case report. It consists of 30 questions, with values equal to or higher than 25 being considered of quality.

For the study of the results, we selected those articles that were of high quality on each scale. For the NOS scale, we analyzed the studies with a score higher than 7, and for the CARE guide, the articles with a score higher than 25.

## 3. Results

**Figure 1:**
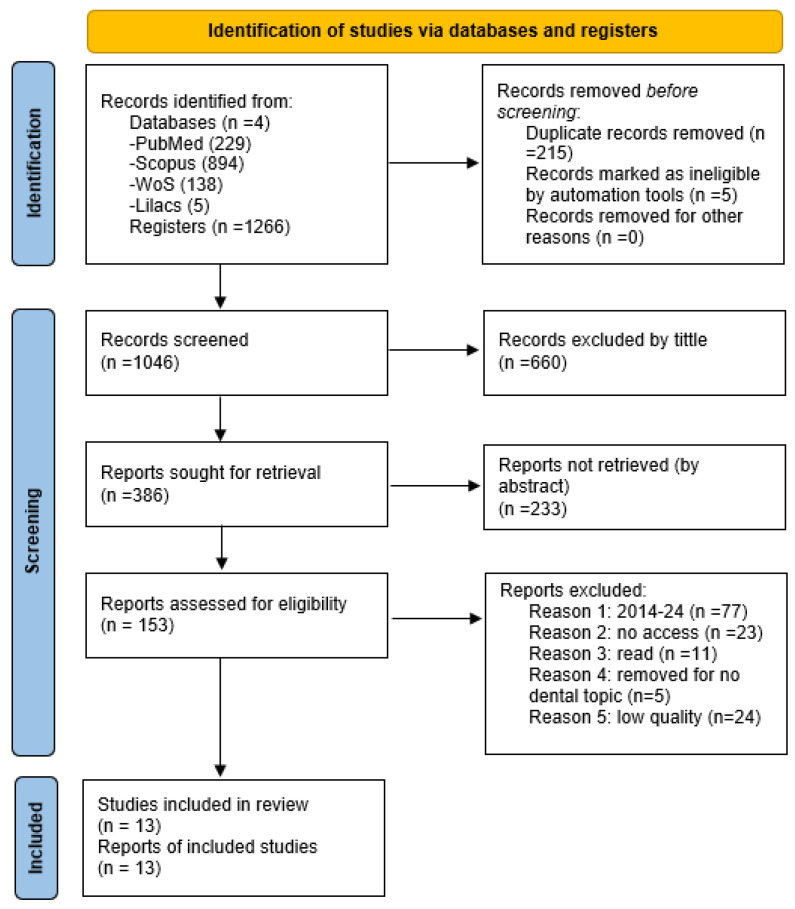
PRISMA statement 2020.

In the search, we found 1266 articles: 894 from Scopus, 229 from PubMed, 5 from Lilacs and 138 from WoS. After removing duplicate items and records marked as ineligible by automation tools, 1046 articles remained: 660 were discarded because of the title, and the abstract of the rest (386) had to be read to determine whether or not they should be included in the review. Finally, 153 articles were selected to be read, but we were unable to access the full text of 23 of them. In total, 140 were discarded after applying the selection criteria, of which only 13 became part of the systematic review (Figure 1, PRISMA diagram).

For the study of the results, we selected those articles that were of high quality on each scale. For the NOS scale (Table 1 and Table 2), we analyzed the studies with a score higher than 7, and for the CARE guide, the articles with a score higher than 25 (Table 3).

**Figure 2:**
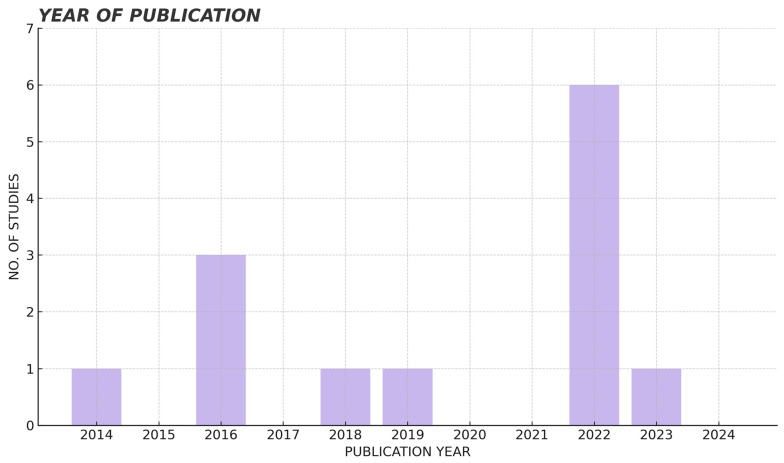
Structure of the studies according to year of publication.

**Figure 3:**
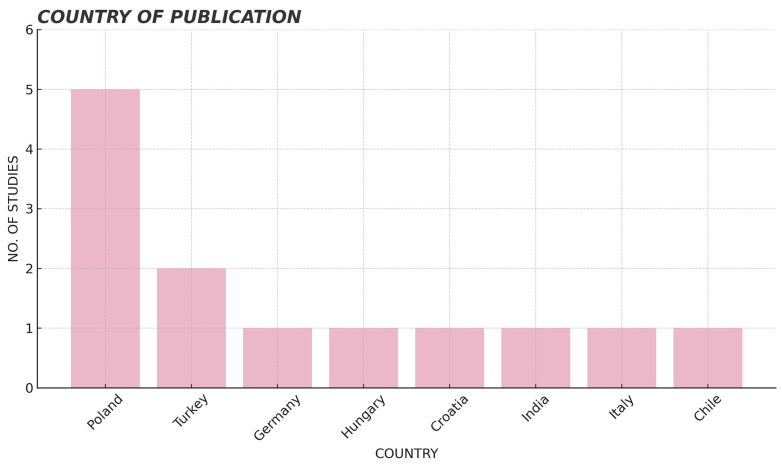
Structure of the studies according to publication country.

The articles are grouped according to year of publication (Figure 2), country of publication (Figure 3) and journal of publication. Regarding the year of publication, studies from the year 2022 stand out, with a total of six articles compared to the rest of the years [16,17,18,19,20,21] (Figure 2). Studies published in Poland predominate [20,22,23,24,25], followed by Turkey [16,26]. Most publications are from European countries (Poland, Hungary, Italy, Croatia and Germany) [19,20,21,22,23,24,27,28,29], followed by the Asian continent (Turkey and India) [16,18,26] and finally the South American continent (Chile) [17] (Figure 3). Regarding the journal of publication, there is a wide variety, being each article of a different journal.

**Table 1:** Quality assessment of case control studies (SCALE NOS).

Study	Type of Condition Studied	Country	Study Design	Criteria								Total Score
				Selection				Comparability	Exposure			
				1	2	3	4	5	6	7	8	
Krasuska-Sławińska et al., 2016 [22].	Congenital defects in permanent teeth	Poland	Cases and controls	*	*	**-**	*	*	*	*	*	**7**
Kilin et al., 2018 [26].	Dental anomalies	Turkey	Cases and controls	*	*	**-**	*	*	*	*	*	**7**
Nemeth et al., 2014 [27].	Late oral sequelae	Hungary	Cases and controls	*	*	*	*	*	*	*	*	**8**
Proc et al., 2016 [23]	Dental complications	Poland	Cases and controls	*	*	**-**	*	*	*	*	*	**7**
Katarzyna Olszewska et al., 2016 [24]	Oral microflora	Poland	Cases and controls	*	*	**-**	*	*	*	*	*	**7**
Cansu Kış et al., 2021 [16]	Mandibular bone	Turkey	Cases and controls	*	*	**-**	*	*	*	*	*	**7**

One * is score.

**Table 2:** Cohort study quality assessment (SCALE NOS).

Study	Type of Condition Studied	Country	Study Design	Criteria								Total Score
				Selection				Comparability	Exhibition			
				1	2	3	4	5	6	7	8	
Longo et al., 2023 [28]	Periodontal tissues	Germany	Retrospective cohort	*	*	*	*	*	**-**	*	*	**7**
Shayani 2021 [17]	Caries and gingivitis	Chile	Retrospective cohort	*	*	*	**-**	*	*	*	*	**7**
Atif et al., 2022 [18]	Dentition	India	Cross-sectional cohort	*	*	*	*	*	**-**	*	*	**7**
Guagnano et al., 2022 [19]	Dentition	Italy	Cross-sectional cohort	*	*	*	**-**	*	*	*	*	**7**
Proc et al., 2019 [29]	Enamel demineralization	Poland	Cross-sectional cohort	*	*	*	**-**	*	*	*	*	**7**
Proc et al., 2022 [20]	Malocclusion	Poland	Cross-sectional cohort	*	*	*	*	*	*	*	*	**8**

One * is one score.

**Table 3:** Quality assessment of case series studies (CARE GUIDELINE).

Articles							Zulijani et al., 2022 [21]					
**Type of condition studied**							**Late dental defect**					
Country							Croatia					
Type of study							Case report.					
Criteria												
Subject	1						*					
Key words	2						*					
Summary	3a	3b		3c		3d	*	*		*		*
Introduction	4						*					
Patient information	5a	5b		5c		5d	*	*		*		*
Clinical findings	6						*					
Timeline	7						*					
Evaluation diagnostic	8a	8b		8c		8d	*	-		*		*
Therapeutic intervention	9a		9b		9c		*		*		-	
Follow-up and results	10a	10b		10c		10d	*	-		-		-
Discussion	11a	11b		11c		11d	*	*		*		*
Patient perspective	12						*					
Informed consent	13						*					
Total							25					

One * is one.

### 3.1. Study Design

Of the 13 articles used for the review, we identify two articles (15.38%) are retrospective cohort [17,28], four (30.76%) are cross-sectional studies [18,19,20,29], six (46.15%) are case control studies [16,22,23,24,26,27], and one (7.69%) is a case report [21] (Appendix A).

### 3.2. Groups or Sample

There is an immense variety in terms of sample size. In the case of cohort studies, both studies divide the samples into study group and control group. In the case of Longo et al. [29], both groups have the same number of patients. However, in the study by Shayani et al. [17], the study group has fewer patients than the control, not making them equivalent (Table A1).

In the case of the case control group, four articles [22,23,24,26] exceed 100 patients, having one of them *n* = 582 patients [23] (Table A2).

In the cross-sectional studies, all exceed 100 patients [18,19,20,29], but only the study by Atif et al. [18] and Guagnano et al. [19] split the sample into equal groups (Table A4).

### 3.3. Age of Participants

Three articles expose the mean age of patients, and one article distinguishes between mean age at the time of treatment and mean age at the time of scan [26]. Three articles distinguish mean age at dental time and mean age at cancer diagnosis [18,19,29]. Two articles provide an age range [24,25], and four articles state a fixed age [20,21,22,27]

### 3.4. Type of Treatment Performed (Chemotherapy, Radiotherapy, Immunotherapy)

Regarding the antineoplastic treatment used, the most frequent is chemotherapy [16,17,18,19,21,22,23,24,25,27], followed by combined therapy with chemo- and radiotherapy [19,26,29]. Two articles describe radiotherapy exclusively [20,23], and only one study includes hematopoietic stem cell transplantation [23] and bone marrow transplantation [19].

### 3.5. Oral/Dental Sequela

There are several oral sequela analyzed among all the selected articles. Two articles analyze the alteration in the oral microbiome [24,28]. These articles study the periodontal status [28] and the occurrence of gingivitis [17]. Four articles analyze the caries index [17,19,27,29]; one article evaluates the alteration in salivary flow [27]; and five articles analyze enamel defects and dental anomalies [18,19,21,22,26]. Finally, one article analyzes the presence of malocclusion after antineoplastic treatment [20], and another analyzes the presence of alterations in the mandibular bone [16].

## 4. Discussion

This systematic review confirms the existence of oral and dental sequelae after antineoplastic treatment in pediatric patients. Their presence affects oral health and function, as well as quality of life, with negative repercussions.

The most used treatment in all studies is chemotherapy, except in the study by Longo et al. [30], Kilin et al. [26] and Guagnano et al. [19], where combined therapy (radiotherapy and chemotherapy) predominated. The study by Guagnano et al. [19] was the only one that included bone marrow transplantation as part of the treatment. For their part, Proc et al. (2016) [23] and Proc (2022) [20] focused their research on patients undergoing exclusively radiotherapy in the head and neck region.

Antineoplastic therapies have different mechanisms of action, so that chemotherapy acts on cell replication, systems eliminating cells with a high rate of cell proliferation, such as cancer cells, as well as healthy cells with high cell growth, such as the oral mucosa [31]. In contrast, radiotherapy has a more localized effect, destroying tumor cells by producing free radicals, acting on cellular DNA and producing collateral damage in tumor as well as healthy cells close to the treated [31]. This fact is going to have relevance in the way oral sequelae manifest themselves in childhood. Patients treated with chemotherapy during childhood are going to have a greater occurrence of dental anomalies and oral mucosal involvement [9,18,21,23,26]. However, those patients treated with radiotherapy are going to have greater involvement in certain areas such as the salivary glands, causing them greater hyposalivation and xerostomia [27,30]. These findings are consistent with previous studies mentioned above such as the reviews by Karen Effinger et al. [3] and Pombo et al. [32], where alterations in dentinogenesis were found. However, the present review provides more updated evidence, integrating recent studies such as that of Longo et al. [30], which represents the first investigation that assesses the alterations that occur at the periodontal level in pediatric patients, and the study by Proc et al. (2022) [20], which studies for the first time the significant association between the presence of dental malocclusion and antineoplastic therapy.

In relation to periodontal alterations, although periodontitis in childhood is unusual [33], antineoplastic treatment is considered a risk factor, as it produces alterations in clinical variables such as periodontal pocket depth and clinical attachment loss [30]. On the other hand, gingivitis is considered the most notorious clinical manifestation in patients undergoing chemotherapy treatments. These data are supported by the studies of Shayani et al. [17] and Alves et al. [10], which coincide in the increase in gingivitis in patients undergoing chemotherapy. Thus, evidence is provided on the correlation between oncological treatment and periodontal complications [10,33].

Radiation affects the salivary glands by modifying their function, damaging the saliva-producing cells. This fact leads to a decrease in salivary flow, known as hyposalivation [27]. The salivary flow has the function of a protective barrier against cariogenic bacteria, preventing the formation of dental caries. In addition, it is responsible for the regulation of oral pH, preventing demineralization of the enamel [27]. Of the articles studied for this review, only the study by Nemeth et al. [27] analyzes the values of salivary flow in the pediatric patient after oncological treatment. As a result, it was obtained that the exposed patients presented a decrease in the salivary flow rate, which continued to be altered at 5 years from the end of treatment, leading to a continuous deterioration of the oral status of these patients. On the other hand, the buffering capacity was lower in the cancer group, which meant a higher risk of caries and enamel demineralization. In addition, children used to quench thirst and dry mouth sensation by consuming foods rich in sugars, which further increased caries risk [34].

Salivary alteration, along with other factors present after antineoplastic treatment, make caries one of the most important sequelae [17,18,26]. As has been previously exposed with the study by Nemeth et al. [27], after treatment, there is an alteration of the buffering capacity of saliva and the regulation of salivary pH, which produces an acidification of the oral environment, damaging the dental structure. To this situation is added the process of dysbiosis that takes place in the oral cavity as a consequence of the increase in cariogenic bacteria such as Lactobacillus and Streptococcus mutans, caused by the lack of oral hygiene and the neutropenia associated with chemotherapy [24].This is a risk factor in altering the microbiota, as documented in the study by Kratazyna Olszewska et al. [24]. The presence of caries was evaluated in the study by Proc et al. [29] with the decayed, missing, filled teeth index (dfmt index for primary teeth) and Decayed, Missing, Filled Teeth index (DMFT) for permanent teeth. An increase in both indexes was determined in cancer patients as being statistically significant in the permanent dentition. In addition, the presence of dental plaque was significantly associated with an increase in the number of decayed and extracted teeth in both primary and permanent dentition. These results are similar to those obtained in the study by Guagnano et al. [19], Nemeth et al. [27] and Shayani et al. [17], where the DMFT and dmft indexes were significantly higher in oncological patients. Similarly, in the study by Shayani et al. [17] that evaluated the caries process over a period of time, it was concluded that the caries index increases with time, being more prevalent at 24 months after completion of treatment.

Odontogenesis may be altered during antineoplastic treatment, resulting in dental alterations [22,23,26]. This fact is detailed in the study by Krasuska-Sławińska et al. [22], where there was a more prevalent existence of enamel defects in the study group than in the controls, such as a higher incidence of dental opacities and hypoplasia. In line with the findings of Krasuska-Sławińska et al. [22], the study by Kılınç et al. [26] associated the age of oncologic treatment and the prevalence of dental alterations. Greater evidence was obtained in the younger age group (children aged 9 months to 4 years), especially in patients with lymphoproliferative tumors in proportion to those with solid tumors. The most affected teeth were the central and lateral incisors and first molars, unlike what was observed by Krasuska-Sławińska et al. [22], where incisors presented less involvement.

On the other hand, regarding the frequency of microdontia, in the research of Kılınç et al. [26], a high prevalence was observed in lateral incisors, first and second premolars and second molars, particularly in the younger age group. These data are different from those contributed by the study of Proc et al. [23] (2016), where a higher frequency of microdontia was demonstrated in second premolars and second molars in cancer patients, whereas in healthy controls, it affected lateral incisors. Similarly, the case report Zulijani et al. [21] showed the presence of microdontia in both maxillary and mandibular first premolars and lateral incisors, which partially differs from the findings of Proc et al. (2016) [23]. Atif et al. [18] also found a significantly elevated prevalence of microdontia in children under 4 years of age, highlighting the importance of age at the time of treatment. It is determined that the stage of dental development and the age at the start of treatment are related, and their influence varies according to the stage of odontogenesis in which the child is [21].

These dental alterations not only compromise dental morphology but can also have a direct impact on occlusion. Proc et al. (2022) [20] assess the correlation between antineoplastic therapy and malocclusion. The modification of the mobility and sensitive function of the tongue and the loss of posterior teeth that cause variations in mastication are the causes of it [35]. In this study, significant differences were observed in some types of malocclusions, such as anterior versus posterior crossbite. Likewise, a lower prevalence of anterior open bite and a reduction in overbite were observed, without the latter being statistically significant. As for dental anomalies, only posterior crossbite and the presence of teeth with short roots in cancer patients were significantly related [20].

However, there are also consequences of antineoplastic therapy in children beyond dental involvement, impacting the quality of bone density in the jawbone [16]. The study by Cansu Kis et al. [16] was the first study to investigate mandibular bone structures in childhood cancer survivors. For this purpose, it used the Klemetti index (KI) in orthopantomograms. It was shown that the cortical bone in this sector presented a C3 category of the KI index, which means a more porous bone and with an endothelium with cortical residues, supporting the hypothesis of a reduction in mandibular bone density induced by antineoplastic treatment [16].

The limitations of this systematic review include potential publication bias, as studies with negative or neutral results may not have been published. While all included studies were of high quality, the predominance of cohort and case control studies limits the ability to generalize findings. Furthermore, the variability in oral sequelae, depending on the specific oncologic treatment and individual factors, adds heterogeneity, which may affect the synthesis and comparison of results.

In view of the above, it is considered that the role of the dentist in the care of these patients is fundamental, both during cancer treatment and during follow-up after its completion. This includes the early diagnosis of acute lesions, the identification of the effects of treatment in the medium/long term and prevention of oral sequelae. In addition, it is very important that this process is carried out in a multidisciplinary way, for which it is essential to focus on oral care from early childhood, as well as establishing protocols for oral treatment and prevention in pediatric patients undergoing oncological treatment.

## 5. Conclusions

The main oral sequelae after oncological treatment in pediatric patients are microdontia, oral mucositis, altered salivary flow, dental caries, enamel alterations, root abnormalities, imbalance of the oral microbiota, affectation of the density of the mandibular bone and dental malocclusion, compared to not-treated children.

- The lesions are different according to the therapy used. In children treated with chemotherapy, the appearance of sequelae related to the alteration of the odontogenesis process is common. On the other hand, children treated with radiotherapy have greater involvement in local areas such as the salivary glands. In addition, there is a correlation between radiotherapy and the presence of dental malocclusion.
- It is not possible to determine which therapy has a greater deleterious effect. It could be said that the most aggressive therapy is chemotherapy because of its systemic effect, while radiotherapy has a field of action more localized. Thus, the combination of both therapies in the studies makes comparison difficult, which could mean a line of research for future studies.

## Data Availability

No new data were created or analyzed in this study. Data sharing is not applicable to this article.

## Appendix A

### Appendix A.1. Cohort Results

**Table A1:** Cohort results. Source: Prepared by the authors.

Author and Year	Type of Study	Participants (No., Age, Type)	Type of Medication	Type of Cancer, Diagnostic Criteria, Type of Tooth Examined and Result Recorded	Results	*p*-Value	Conclusion
Longo et al., 2023 [28]	Retrospective cohort	No. of participants: 72 GS: 36 (cancer patients) GC: 36 (healthy patients) Age: 12.0 ± 3.9/4.0	Chemotherapy (QT): *n* = 36 Chemotherapy (QT) and radiotherapy (RT): *n* = 9	Type of cancer: lymphoid leukemia, myelodysplastic syndrome, Hodgkin’s lymphoma, Non-Hodgkin’s lymphoma, Burkitt’s lymphoma, intracranial and intraspinal cancer, renal carcinoma, gonadal carcinoma, rhabdomyosarcoma, other sarcomas Diagnostic criteria: - Saliva samples: cPCR and DNA extraction - Oral examination: periodontal probe Hygiene: IP, GI, CAL Type of tooth examined: permanent dentition Recorded result: periodontal status and oral microbiome	Saliva samples *Porphyromonas gingivalis* - GS: 0.00 (0.00–0.00) - GC (: 0.00 (0.00–0.00) *Aggregatibacter actinomycetemcomitans* - GS: 3.64 (3.39–3.80) - GC: 3.66 (3.02–4.03) *Tannerella forsythia* - GS: 0.00 (0.00–1.60) - GC: 1.09 (0.00–1.44) *Fusobacterium nucleatum* - GS: 2.55 (2.11–2.78) - GC: 2.22 (1.91–2.49) Clinical parameters IP CC: 30.5% (5–73)/GC: 22.6% (2–61) **IG** CC 28.8% (5–60)/GC 17.3% (2–44) **PS** CC 1.77 (1.43–2.43)/GC 1.61 (1.30–2.09) **CAL** CC 1.77 (1.42–2.44)/GC 1.57 (1.21–2.09) **CAL ≥ 4 mm** CC 28 GC 16 **Periodontal status** Periodontal health: CC 3 GC 7 Gingivitis CC 16 GC 24 Periodontitis CC 17 GC 5	*p* = 0.33 *p* = 0.37 *p* = 0.29 *p* = 0.02 *p* = 0.02 *p* ≤ 0.001 *p* ≤ 0.008 *p* ≤ 0.001 *p* ≤ 0.001	Antineoplastic therapy in cancer patients has a negative impact on periodontal and microbiological clinical parameters.
Shayani et al., 2022 [17]	Retrospective cohort	**No. of participants:** 69 **GS (Study group):** 23 **GC (control group):** 46 **Age:** 3.25–12.9 years)	QT	**Type of cancer:** acute lymphoblastic leukemia **Diagnostic criteria:** - ALL IC-BFM Chemotherapy Protocol 2009 - **New** caries lesions: caries index described by WHO - Caries history: number of teeth decayed, lost due to caries or filled - Gingivitis: if present/absent **Type of tooth examined:** permanent dentition **Recorded outcome:** dental caries, presence of new lesions during treatment and gingivitis	Clinical parameters: **Caries index:** Exposed: [4.48 (SD 3.51)] Not exposed: [3.01 (SD 2.92)] **DMFT index:** Exposed:1.58 (SD 2.87) Unexposed: 0.31 (SD 0.64) **No. of teeth with presence of new lesions during treatment** Exposed: 60.87% Not exposed: 28.26% **Gingivitis** Exposed: 47.83%; −69.57% Not exposed: 84.78%	*p* = 0.54 ***p* < 0.05** ***p* = 0.008** ***p* = 0.001**	The ALL IC-BFM 2009 chemotherapy protocol in patients with acute lymphoblastic leukemia is a risk factor in the development of new caries and gingivitis lesions.

### Appendix A.2. Results of Cases and Controls

**Table A2:** Results cases and controls.

Author and Year	Type of Study	Participants (No., Age)	Type of Medication	Type of Cancer, Diagnostic Criteria, Type of Tooth Examined and Result Recorded	Results	*p*-Value	Conclusion
Krasuska-Sławińska et al., 2016 [22]	Cases and controls	No. of participants: 120 GS: 60 (cancer patients) GC:60 (healthy patients) Age: <18	Chemotherapy Drugs VCR, CTX, ADM, VP-16, CDDP, IF, ACTD	Diagnosis: Burkitt’s lymphoma (15.0%), nephroblastoma (13.0%), neuroblastoma (10.0%), histiocytosis (8.3%), rhabdomyosarcoma (6.7%), Ewing’s disease sarcoma (6.7%), medulloblastoma (5.0%), neurofibromatosis type I (5.0%) and others (19.7%) Criteria: Clinical OPG: evaluation of congenital syndromes modified DDE index (opacities, hypoplasia): size and anatomical shape of the crown and enamel defects Höltta defect index: evaluates quantitative and qualitative anomalies Root length: C/R ratio Data analysis: Mann Whitney U and Spearman’s RHO metric test Type of tooth examined: permanent dentition Recorded result: dental alterations	GS CG Enamel defects: 53/88.3 24/40.0 Opacities: 34/56.6 19/32.3 Hypoplasia: 7/11.6 1/1.6 Combination of injuries: 12/20 4/6.6 Tooth resorption: 36/60.0 25/41.6 V-shaped: 18/30.00 0/0.0 Microdontia: 12/21.67 0/0.0 Without dental yolk: 16/26.67 4/6.66 From >0: 45/75.00 2643.33 Others: Taurodontism: 12/20.00 3/5.00 Mesiodens: 1/1.67 0/0.0 Dentinoma: 18/30.0 9/15.0 Impacted teeth: 6/10.00 2/3.33	*p* = 0.000 ***p* = 0.006** ***p* = 0.001** ***p* = 0.0068** ***p* = 0.0450** ***p* = 0.000** ***p* = 0.0003** ***p* = 0.0035** ***p* = 0.0004** ***p* = 0.0135** *p* = 0.3254 *p* = 0.0505 *p* = 0.1466	Chemotherapy in the pediatric setting affects the appearance of new congenital dental alterations; hypodontia, microdontia, root resorption, taurodontism and congenital enamel defects. The drugs used in chemotherapy have a negative influence on the dental level, and their effect is more detrimental with the increase in the dose, the duration of therapy and the severity of the effects derived from chemotherapy such as mucositis and vomiting.
Kilin et al., 2018 [26]	Cases and controls	Total number of participants: 165 GS: Group A (9 months–4 years): 59 Group B (5–7 years): 34 *Average age of treatment:* 3.75 + −2.01 *Mean age at examination:* 9.54 + −1.25 (8–13 years) GC 72 Average age: 10.60 + −2.40 (8–16 years)	QT and RT	Type of cancer: leukemia, lymphoma, neuroblastoma, renal tumor, retinoblastoma, liver tumor, soft tissue sarcoma, germ cell tumor, Langerhans cell histiocytosis Diagnostic criteria: OPG, intraoral examination Hölttä disturbance index: evaluation of root length Enamel defect (ED): hypoplasias > 2 mm Type of tooth examined: permanent dentition Recorded result: dental alterations	GS (A and B): Hypodontia 21 (22.6) Microdontia 60 (64.5) Root malformations 24 (25.8) Enamel defects 22 (23.7) Hyperodontia 1 (1.1) GC Enamel defects: 7 (9.7) Comparison of patient groups according to age of treatment. Group A Group B Hypodontia 17 (28.8) 4 (11.8) Microdontics 42 (71.2) 18 (52.9) Malformation radicular 13 (22.0) 11 (32.4) Enamel defects 14 (23.7) 8 (23.5) Hyperodontia 1 (1.7) 0 (0.0)	*p* = 0.058 *p* = 0.077 *p* = 0.273 *p* = 0.982 *p* = 1.000 *p* = 0.982 *p* = 1.000 *p* = 0.058 *p* = 0.077 *p* = 0.273 *p* = 0.982 *p* = 1.000	Patients treated with chemotherapy before the age of 7 years constituted a high-risk group for dental anomalies. The frequency of microdontia and hypodontia increased even more when the patient received oncologic treatment before the age of 5 years.
Nemeth et al., 2014 [27]	Cases and controls	Total number of participants: 78 GS: 38 GC: 40 (healthy) Age: 12 years old	QT	Type of cancer: lymphoblastic lymphoma, neuroblastoma, sarcoma, osteosarcoma, Hodgkin’s lymphoma Diagnostic criteria: Clinical examination: World Health Organization guidelines Salivary examination: Sreebny method Caries index: DMFT for permanent dentition Type of tooth examined: permanent dentition Recorded outcome: long-term oral effects of chemotherapy and correlation between salivary flow and karyological status	caries index: Patients with QT (GS) No. decayed teeth (C, O): 3.97 ± 3.58 vs. 0.84 ± 1.75, *p* ≤ 0.001 No. of teeth affected (DMFT): 4.61 ± 3.71 Healthy patients (CG) No. decayed teeth (A, O): 0.58 ± 1.34 vs. 1.18 ± 1.13, *p* ≤ 0.05) No. of teeth affected (DMFT): 2.21 ± 2.10 Stimulated and unstimulated total saliva flow rates Patients with QT (GS) SSF: 0.85 ± 0.49 USF: 0.28 ± 0.26 PS: 1.643 ± 2.42 Buffer capacity: 0 (low) 18.4 (medium) 81.6 (high) Healthy patients (CG) SSF: 1.13 ± 0.48 USF: 0.378 ± 0.24 PS: 0.456 ± 0.32 Buffer capacity: 2.5 (low) 57.4 (average) 40 (high) Correlation between USF, SSF, PS and DMFT GS USF SSF PS DMFT −0.32 −0.35 −0.49 GC USF SSF PS (no data)	*p* = 0.01 *p* = ≤0.05 *p* ≤ 0.1 *p* ≤ 0.001 *p* ≤ 0.01 *p* ≤ 0.01 (*p* ≤ 0.01) GS USF: *p* = 0.02 SSF: *p* = 0.02 PS: *p* = 0.001	Chemotherapy in children could cause a decrease in total stimulated salivary flow, hyposalivation and, consequently, an increased risk of caries.
Proc et al., 2016 [23]	Case and controls	Total number of participants: 582 Study group: 61 Control group: 521 (healthy) Age: 5–18	RT in head and neck	Type of cancer: lymphoblastic leukemia, acute non-lymphoblastic leukemia, B-cell non-Hodgkin’s lymphoma, Hodgkin’s lymphoma, germinal tumor, brain tumor, Wilms’ tumor, hepatoblastoma, neuroblastoma, primitive neuroectodermal tumor or rhabdomyosarcoma Diagnostic criterion: OPG Type of tooth examined: permanent dentition Recorded result: dental anomalies	Dental agenesis SG CC IC 4 (1.63) 5 (0.23) IL 4 (1.63) 28 (1.34) C 2 (0.81) 10 (0.47) 1PM 1 (0.4) 9 (0.43) 2PM 31 (12.7) 33 (1.58) 1M 4 (1.63) 4 (0.19) 2M 23 (9.42) 2 (0.095) 3M NA Healthy teeth 175 (71.72) 1993 (95.63) Microdontia SG CC IC 0 0 IL 0 14 (0.67) C 0 1 (0.047) 1PM 16 (6.55) 1 (0.047) 2PM 25 (10.24) 0 1M 0 0 2M 24 (9.83) 2 (0.095) 3M 12 (4.91) 2 (0.095) Healthy teeth 167 (68.44) 2064 (99.04) Root shortening CI 0 7 (0.33) IL 0 6 (0.28) C 0 4 (0.19) 1PM 4 (1.63) 5 (0.23) 2PM 8 (3.27) 4 (0.19) 1M 14 (5.73) 4 (0.19) 2M 12 (4.91) 6 (0.28) 3M 0 0 Healthy teeth 206 (84.42) 2048 (98.27)	*p* = 0.001 *p* = 0.712 *p* = 0.478 *p* = 0.945 *p* = 0.001 *p* = 0.001 *p* = 0.001 *p* = 0.001 - *p* = 0.199 *p* = 0.734 *p* = 0.001 *p* = 0.001 - *p* = 0.001 *p* = 0.001 *p* = 0.001 *p* = 0.368 *p* = 0.407 *p* = 0.495 *p* = 0.001 *p* = 0.001 *p* = 0.001 *p* = 0.001 *p* = 0.001	Children under 5 years of age are in a high-risk group for dental complications following cancer treatment. Basic chemotherapy has a considerable impact on the occurrence of dental anomalies.
Katarzyna Olszewska et al., 2016 [24].	Case and controls	Total number of participants: 104 GS: 52 (37 patients with hematologic cancer and 15 patients with solid tumors) GC: 52 healthy patients Age: 3–17.5a	QT Medication (not specified)	Type of cancer: leukemia, lymphoma and solid tumors Diagnostic criteria: *Streptococcus mutans* evaluation: Dentocult SM Strips Mutans tests (Orion Diagnostica) Evaluation No. of *Lactobacillus* spp: Dentocult LB tests (Orion Diagnostica, Espoo, Finland) Results analysis: STATISTICA 10 of Windows Software (StatSoft Inc., Tulsa, OK, USA) Type of teeth examined: permanent and deciduous dentition Recorded result: no. of *Lactobacillus* sp. and *Streptococcus mutans*	Evaluation no. of *Streptococcus mutans:* 0 1 2 3 GS T1 11 28 12 1 T2 7 11 19 15 T3 8 16 25 3 GC 12 20 16 4 Evaluation nº of *Lactobacillus* spp: 0 10^3^10^4^10^5^10^6^ CFU/mL SG T1 14 21 15 1 1 1 T2 9 8 12 15 8 T3 7 15 21 8 1 CG 11 22 11 5 3	*Streptococcus mutans* *p* = T1:0.29 *p* = T2:0.0144 * *p* = T3: 0.3389 *Lactobacillus* spp. *p* = T1: 0.3234 *p* = T2:0.0071 * *p* = T3:0.1280	The alteration of the bacterial flora presents changes and is statistically related to the degree of neutropenia induced by chemotherapy.
Cansu kis et al., 2022 [16]	Case and controls	Total number of participants: 98 GS: 49 GC: 49 (healthy) Age: 14.5 ±4.4 years and 14.6 ±4.8 years	QT (dexametha- sone; prednisolone; methotrexate)	Diagnosis: Hodgkin’s lymphoma, non-Hodgkin’s lymphoma, Burkitt’s lymphoma, acute lymphoblastic leukemia, brain tumor, osteosarcoma, neuroblastoma Diagnostic criteria: OPG Facial dimension analysis: White and Rudolph box-counting method Classification scale according to cortical bone appearance according to KI Type of teeth examined: permanent and deciduous dentition Recorded result: alterations of the mandibular bone	Categories Klemetti index (KI) C1 GS:21 (42.3) GC:33 (67.3) C2 GS:24 (49.0) GC:16 (32.7) C3 GS:4 (8.7) GC:0 (0.0)	Statistically significant difference in C3 study group *p* = 0.015 *	Chemotherapy is related to the affectation of the mandibular bone structures in the long term in the patient’s life. The Klemetti Index is considered a good tool for the clinical diagnosis of mandibular bone conditions.

Abbreviations cases and controls: ACTD = actinomycin; ADM = doxorubicin; C = canine; CTX = cyclophosphamide; CDDP = cisplatin; ED = enamel defects; GS = study group; GC = control group; IC = central incisor; IF = ifosfamide; IL = lateral incisor; KI = Klimetti index; M = molar; OPG = orthopantomography; PS = palatal salivary flow; PM = premolar; SSF = stimulated total salivary flow; US = unstimulated total salivary flow; VCR = vincristine; VP-16 = etoposide. * means it is statistically significant.

### Appendix A.3. Results of Case Reports

**Table A3:** Results of case reports. Source: own elaboration.

Author and Year	Type of Study	Participants (No., Age)	Type of Medication	Diagnosis, Criteria, Type of Tooth Examined and Result Recorded	Results	*p*-Value	Conclusion
Zulijani et al., 2022 [21]	Case Report	Total number of participants: 1 Age: 9	QT Drugs: cyclophosphamide vincristine, methotrexate, etoposide and carboplatin	Diagnosis: Anaplastic Ependymoma grade III Criteria: intraoral and extraoral examination, OPG Teeth examined: primary and permanent dentition (16, 55, 54, 11, 21, 63, 64, 65, 26, 36, 75, 74, 33, 32, 31, 41, 42, 43, 84, 85 and 46) Recorded result: dental anomalies	Mucositis Microdoncia: 12, 14, 16, 22, 24, 26, 32, 34, 36, 42, 44, 46	Not described	There is an alteration of dental development in children treated with antineoplastic therapy, so dentists should keep in mind that these patients should be followed up after the end of treatment to reduce the consequences of the treatment.

Abbreviations case report: QT = chemotherapy; OPG = orthopantomography.

### Appendix A.4. Results of Cross-Sectional Studies

**Table A4:** Results of cross-sectional studies. Source: own elaboration.

Author and Year	Type of Study	Participants (No., Age)	Type of Medication	Diagnosis, Criteria, Type of Tooth Examined and Result Recorded	Results	*p*-Value	Conclusion
Atif et al., 2022 [18]	Cross-sectional	No. of participants: Study Group (GS): 120 Control group (CG): 121 Mean age at dental examination: 14.3 (GS)–14.4 (GC) years Mean age (cancer diagnosis): 5.67 years	Chemotherapy (not specified) QT Home (i) <4a (ii) 4–5a (iii) 6–7a	Type of cancer: acute lymphoblastic leukemia (45%), soft tissue sarcoma, Hodgkin’s lymphoma, medulloblastoma, Langerhans cell histiocytosis, Retinoblastoma, primitive neuroectodermal tumor, Non-Hodgkin’s lymphoma, Osteosarcoma Diagnostic criteria: - Oral examination: mirror and CPITN probe - Microdontics: king’s foot (anterior teeth) - compass (posterior teeth) - Absences: clinical examination - Enamel effects: modified enamel index Type of tooth examined: permanent dentition Recorded result: microdontia, hypodontia, teeth with abnormal anomaly and enamel development defects	Study Group (GS): Microdontia: 21 (17.5%) Hypodontia 6 (5%) Abnormally shaped teeth: 10 (8.33%) Enamel developmental defects: 45 (37.5%) Control group (CG): Microdontia: 10 (8.2%) Hypodontia: 3 (2.5%) Abnormally shaped teeth: 2 (1.65%) Enamel developmental defects: 27 (22.3%) No presence of any anomaly: GS: 57 GC: 82 Correlation between age of onset and presence of dental anomalies Microdontia <4 years (*n* = 22): 40.9% 4–5 years (*n* = 40): 15% >6 years (*n* = 58): 10.3% Hypodontia <4 years (*n* = 22): 9.1% 4–5 years (*n* = 40): 0% >6 years (*n* = 58): 6.9% Abnormally shaped teeth <4 years (*n* = 22): 9.1% 4–5 years (*n* = 40): 10% >6 years (*n* = 58): 6.9% Enamel defects <4 years (*n* = 22): 40.9% 4–5 years (*n* = 40): 45% >6 years (*n* = 58): 31%	Microdontia: 0.032 Hypodontia: 0.302 Abnormally shaped teeth: 0.017 Defects of enamel development: 0.01 *p* = 0.005 * *p* = 0.106 *p* = 0.853 *p* = 0.349	Statistically significant association between dental anomalies and antineoplastic therapy, with prevalence of microdontia, teeth with abnormal appearance and enamel defects among childhood cancer survivors.
Guagnano et al., 2022 [19]	Cross-sectional	No. of participants: 104 Study group (GS): 52 Control group (CG): 52 (GS) Age at time of dental examination: 10.6 ± 3.8 years (GC): 11.5 ± 4.5 years Age of diagnosis of cancer: 3.8 ± 2.6 years	Chemotherapy (*n* = 36) Chemotherapy and radiotherapy (*n* = 16) Hematopoietic stem cell/bone marrow transplantation (*n* = 19/5)	Type of cancer: acute lymphoblastic leukemia, acute myeloblastic leukemia, medulloblastoma, familial hematological lymphohistiocytosis, lymphoma, juvenile myelomonocytic leukemia, Wilms tumor, hepatoblastoma, rhabdomyosarcoma, Ewing sarcoma-PNET, severe aplastic anemia, Xanthoastrocytoma, histiocytosis, anaplastic broad cell lymphoma. Diagnostic criteria: Caries index: dmft index (primary teeth) DMFT Index (permanent teeth) Alterations of enamel mineralization: Aine classification scale Dental anomalies: OPG Lind method: root to crown ratio (R/C) Höltta defect index NSAID classification Dental age: Demirjian Method [Demirjian, Goldstein, Tanner, 1973] Type of tooth examined: permanent dentition Recorded result: dental alterations	Dental anomalies *Defects in dental development according to the Defects Index in the study groups* GS GC D1 3.57 ± 4.26 ** 0.85 ± 2.16 D2 0.74 ± 2.17 ** 0.00 D3 0.19 ± 0.50 ** 0.00 D4 1.91 ± 3.16 ** 0.00 D5 0.94 ± 1.75 *** 0.29 ± 0.71 <5 years >5 years D1 2.94 ± 4.16 5.07 ± 4.27 * D2 0.97 ± 2.56 0.21 ± 0.43 D3 0.24 ± 0.56 0.07 ± 0.27 D4 2.64 ± 3.53 ** 0.21 ± 0.58 D5 1.30 ± 1.98 * 0.07 ± 0.27 QT QT + RT Transplant D1 2.56 ± 3.07 5.73 ± 5.61 * 4.05 ± 4.81 D2 0.34 ± 1.12 1.60 ± 3.40 1.45 ± 3.04 * D3 0.13 ± 0.42 0.33 ± 0.62 0.36 ± 0.66 * D4 1.53 ± 2.58 2.73 ± 4.13 3.59 ± 3.94 *** D5 0.81 ± 1.49 1.20 ± 2.24 1.82 ± 2.24 *** Enamel defects *NSAID classification* CCS GC AINE 1 3.36 ± 5.31 *** 0.19 ± 0.71 AINE 2 0.64 ± 1.48 *** 0.04 ± 0.29 AINE 3 0.56 ± 1.47 *** 0.00 AINE 4 0.27 ± 0.86 *** 0.00 Caries rate dmft DMFT CCS: 4.15 ± 3.25 * 1.70 ± 2.21* * * * GC: 2.59 ± 3.04 1.09 ± 1.79 Males: 3.83 ± 3.56 3.46 ± 3.89 *. Females: 6.07 ± 5.27 1.75 ± 2.92 <5 years old: 4.87 ± 4.82 1.90 ± 3.25 > 5 years: 4.00 ± 1.85 4.40 ± 3.72 * 4.40 ± 3.72 * 4.40 ± 3.72 * 4.40 ± 3.72 * 4.00 ± 1.85 QT: 4.57 ± 4.23 2.58 ± 3.16 QT + RT: 5.11 ± 5.06 3.00 ± 4.41 Transplantation: 4.00 ± 3.73 1.52 ± 3.30 Non-transplant: 5.35 ± 4.91 3.72 ± 3.54 *** 3.72 ± 3.54 *** 3.72 ± 3.54 *** 3.72 ± 3.54	* *p* < 0.05 ** *p* < 0.01 *** *p* < 0.001 Root malformations, agenesis and microdontia (*p* < 0.01) Enamel defects: *p* <0.001) *p* = 0.029	These children are at high risk for dental developmental abnormalities and poor dental health and should be closely supervised by a specialist dentist.
Proc et al., 2019 [29]	Cross-sectional	No. of participants: 225 GS: 75 GC: 150 Age: 4–18 years old	QT RT	Type of cancer: acute lymphoblastic leukemia, Williams tumor, neuroblastoma, rhabdomyosarcoma, brain tumor, hepatoblastoma, acute non-lymphoblastic leukemia, non-Hodgkin’s lymphoma, Hodgkin’s lymphoma, primitive neuroectodermal tumor, germ cell tumor, ovarian tumor Diagnostic criteria Caries rate - dmft index in primary teeth - DMFT index in permanent teeth (number of decayed + missing + filled teeth) Oral hygiene: IP of Silness-Löe Cancer risk: questionnaire Type of neoplasm on the incidence of caries Teeth examined: permanent and deciduous dentition Recorded result: caries and dental plaque index; Influence of radiotherapy and caries prevalence	caries index: dmft: GS 5.02 ± 4.1/GC 4.14 ± 2.94 DMFT: GS 2.60 ± 3.14/GC 1.59 ± 2.79 Correlation between oral hygiene and caries index: IP <1 dmft 25 DMFT 32 >1 dmft 25 DMFT 36 Influence between radiotherapy and the prevalence of caries. Variable No RTX with RTX d 3 (0.5–6.5) 2.5 (1–4) m 0 (0–0) 0 (0–0) f 0 (0–1) 2 (1–3) dmft 4 (1–8) 5 (4–6) D 0 (0–2) 2 (0–3) M 0 (0–0) 0 (0–0) F 0 (0–2) 1.5 (0–2) DMFT 2 (0–4) 4.5 (1–6)	*p* = 0.3722 *p* = 0.0053 dmft *p* = 0.0914 DMFT *p* = 0.0252 *p* = 0.762965 *p* = 0.747707 *p* = 0.012871 *p* = 0.499063 *p* = 0.043573 *p* = 0.574069 *p* = 0.230553 *p* = 0.048725	Childhood cancer patients have an increased risk of advanced dental caries that could be prevented by oral health education.
Proc et al., 2022 [20]	Cross-sectional	No. of participants: 225 GS: 75 GC: 150 Age: 16 Dental examination: 6–155 months (mean 4.9 years) after cessation of antineoplastic treatment when patients were aged between 47 months (4 years) and 215 months (18 years)	Head and neck radiotherapy	Type of cancer: leukemia, B-cell non-Hodgkin’s lymphoma, Hodgkin’s lymphoma lymphoma, peripheral primitive neuroectodermal tumor, germinal tumor, and ovarian tumor Diagnostic criteria: - Intraoral examination by two examiners - Instruments: flashlight, a mouth mirror and a metal millimeter ruler - Data collection: WHO form - Occlusal evaluation: centric relation and maximum intercuspidation - Anteroposterior relations: Angle class I, II and III Type of tooth examined: permanent dentition Recorded outcome: dental malocclusions in relation to antineoplastic treatment	GS/GC Malocclusion 49 (65.33%)/99 (65.56%) Class I 51 (68.00%)/94 (62.25%) Class II (dystocclusion) 21 (28.00%)/52 (34.44%) Class III (inversion of horizontal protrusion) 6 (8.00%)/3 (1.99%) Abnormal overjet (incisal protrusion) 3 (4.00%)/5 (3.31%) Anterior crossbite 10 (13.33%)/6 (3.97%) Posterior cross bite 14 (18.67%)/7 (4.64%) Anterior open bite 1 (1.33%)/13 (8.61%) Posterior open bite 2 (2.67%)/0 Deep bite 1 (1.33%)/12 (7.95%) Scissor bite 0 2 (1.32%) Overcrowding/rotations 6 (8.00%)/15 (9.93%) Misalignment 14 (18.67%)/12 (7.95%) Diastemas 2 (2.67%)/0 Midline shift/jaw shift 3 (4.00%)/6 (3.97%) Maxillary compression 1 (1.33%)/11 (7.28)	*p* = 0.9727 *p* = 0.9361 *p* = 0.3670 *p* = 0.0628 *p* = 1.0000 *p* = 0.0136 *p* = 0.0012 *p* = 0.0387 *p* = 0.1091 *p* = 0.0651 *p* = 1.0000 *p* = 0.8195 *p* = 0.0310 *p* = 0.1091 *p* = 1.0000 *p* = 0.0662	Oncological treatment may alter the development of occlusion in cancer patients.

Abbreviations cross-sectional studies: A = absences; C = caries; GS = study group; CG = control group; PI = plaque index; ND = not determined; O = obturations; QT = chemotherapy.
